# Food allergy and anaphylaxis – 2049. Evolution of food allergy in a high risk population: the Canadian asthma primary prevention study (CAPPS)

**DOI:** 10.1186/1939-4551-6-S1-P133

**Published:** 2013-04-23

**Authors:** Rishma Chooniedass, Brenda Gerwing, Saiful Huq, Clare Ramsey, Anita Kozyrskyj, Edmond Chan, Moira Chan-Yeung, Allan Becker

**Affiliations:** 1 Pediatrics and Child Health/ Section of Allergy, University of Manitoba, Canada; 2 Internal Medicine, University of Manitoba, Winnipeg, MB, Canada; 3 Pediatrics, University of Alberta, Edmonton, AB, Canada; 4 Pediatrics, University of British Columbia, Vancouver, BC, Canada; 5 Internal Medicine, University of British Columbia, Vancouver, BC, Canada

## Background

Food allergy is on the rise. It is often assumed that allergy in early life to milk and egg often resolves whereas this is less frequent with peanut.

## Methods

CAPPS is a high risk allergy and asthma birth cohort. 545 families were enrolled during pregnancy in Winnipeg and Vancouver, Canada. Study participants were prenatally randomized into a multifaceted modified diet, lifestyle and environment intervention group or control group. Questionnaires were completed prenatally and when the children were assessed by a Pediatric Allergist at 1, 2, 7 and 15 years of age. Assessments included skin testing to common inhalant and ingestant (milk, egg and peanut) allergens. A positive skin test was defined as having a mean wheal diameter ≥ 3mm.

## Results

At age 1, 3.4% (16/474) of children were sensitized to milk, 9.1% (43/474) to egg and 5.3% (25/474) to peanut. At age 15, 1.6% (5/321) were sensitized to milk, 1.9% (6/321) to egg and 10.9% (35/321) peanut. At age 15, 100% of children sensitized to milk and egg at age 1 were no longer sensitized to those foods. Interestingly 64% (16/25) of the children sensitized to peanut at age 1 outgrew sensitization to peanut at age 15. New food sensitizations developed between the ages of 1 and 15. Sensitization to peanut at age 1 does have an increased risk of sensitization to peanut at age 15 (OR=9.4, 95% CI 3.6-25.0). However, sensitization to peanut at age 2 has the greatest likelihood of persistence (OR=35.8, 95% CI 14.0-91.9). At age 15, 5.6% of those tested (18/322) had developed sensitization to peanut after age 7 while 3% (10/322) of those sensitized at age 7 to peanut were no longer skin test positive. Similarly from age 7 to 15, 1.6% (5/322) became sensitized to milk and 1.6% (5/322) became sensitized to egg.

## Conclusions

Food sensitization to milk, egg and peanut decrease over time. The greatest likelihood for persistent peanut sensitization is seen with a positive skin test at age 2. Risk factors for new sensitization and factors associated with the loss of sensitization need to be defined.

